# 

**Published:** 1893-03-25

**Authors:** 


[Registered at the General Post Office as a Newspaper for transmission in the United Kingdom and Abroad.]

WITH EXTRA NURSING SUPPLEMENT
Per Annum, 8s. 6d., post free.
Monthly Parts, 6d.; post free, 8d.
Ditto per Annum; 8s., post free.

### THE HOSPITAL

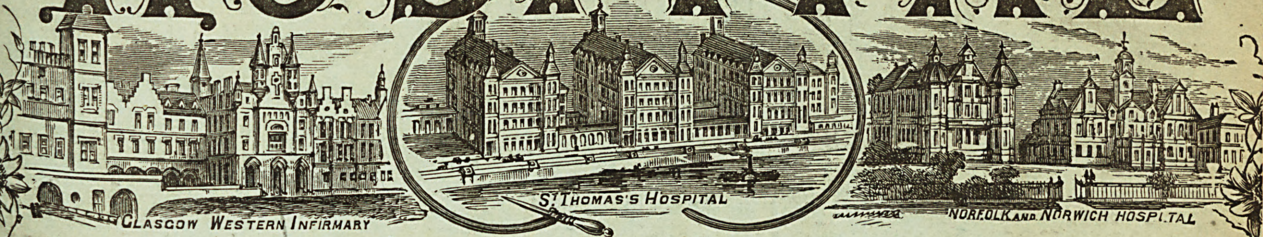

WITH EXTRA NURSING SUPPLEMENT.

No. 339. Vol. XIII.] SATURDAY, MARCH 25th, 1893. [TWOPENCE.

